# Focused Ultrasound (FUS) for Chronic Pain Management: Approved and Potential Applications

**DOI:** 10.1155/2021/8438498

**Published:** 2021-06-29

**Authors:** Lazzaro di Biase, Emma Falato, Maria Letizia Caminiti, Pasquale Maria Pecoraro, Flavia Narducci, Vincenzo Di Lazzaro

**Affiliations:** ^1^Unit of Neurology, Neurophysiology, Neurobiology, Department of Medicine, Università Campus Bio-Medico di Roma, Via Álvaro del Portillo 21, Rome 00128, Italy; ^2^Brain Innovations Lab, Università Campus Bio-Medico di Roma, Via Álvaro del Portillo 21, Rome 00128, Italy

## Abstract

Chronic pain is one of the leading causes of disability and disease burden worldwide, accounting for a prevalence between 6.9% and 10% in the general population. Pharmacotherapy alone results ineffective in about 70-60% of patients in terms of a satisfactory degree of pain relief. Focused ultrasound is a promising tool for chronic pain management, being approved for thalamotomy in chronic neuropathic pain and for bone metastases-related pain treatment. FUS is a noninvasive technique for neuromodulation and for tissue ablation that can be applied to several tissues. Transcranial FUS (tFUS) can lead to opposite biological effects, depending on stimulation parameters: from reversible neural activity facilitation or suppression (low-intensity, low-frequency ultrasound, LILFUS) to irreversible tissue ablation (high-intensity focused ultrasounds, HIFU). HIFU is approved for thalamotomy in neuropathic pain at the central nervous system level and for the treatment of facet joint osteoarthritis at the peripheral level. Potential applications include HIFU at the spinal cord level for selected cases of refractory chronic neuropathic pain, knee osteoarthritis, sacroiliac joint disease, intervertebral disc nucleolysis, phantom limb, and ablation of peripheral nerves. FUS at nonablative dosage, LILFUS, has potential reversible and tissue-selective effects. FUS applications at nonablative doses currently are at a research stage. The main potential applications include targeted drug and gene delivery through the Blood-Brain Barrier, assessment of pain thresholds and study of pain, and reversible peripheral nerve conduction block. The aim of the present review is to describe the approved and potential applications of the focused ultrasound technology in the field of chronic pain management.

## 1. Introduction

Pain is the unpleasant sensory and emotional experience associated with, or resembling that associated with, actual or potential tissue damage. Acute pain is associated with tissue damage, inflammation, or disease of a relatively brief duration. Pain lasting longer than three months is defined as chronic pain, according to the International Association for the Study of Pain (IASP) [1]. Three categories of chronic pain could be distinguished: nociceptive pain, as a consequence of direct tissue disease or damage; neuropathic pain caused by somatosensory system disease or damage; and mixed pain, meaning the combinations of both nociceptive and neuropathic pain [2]. Worldwide, chronic pain is one of the leading causes of disability and disease burden. According to the Global burden of Disease Study 2016, the most common symptomatic chronic condition is recurrent tension-type headaches, affecting 1.9 billion people, while low back and neck pain are the leading causes of disability internationally [3]. Chronic neuropathic pain prevalence ranges between 6.9% and 10% in the general population [4]. The World Health Organization (WHO) suggests a three-step ladder to guide analgesic medication therapy. The ladder consists of a first step of oral given nonopioid medication, such as aspirin, paracetamol, or other nonsteroidal anti-inflammatory drugs (NSAID), at increasing dosage until pain relief is reached. If ineffective, the second step includes the adjunction of weak opioid for mild to moderate pain. The third level of the ladder calls for a strong opioid for moderate to severe pain (e.g., morphine). Adjuvants medications (anxiolytics, hypnotics, and muscle relaxants) can be added at any step of the ladder [5]. Concerning neuropathic pain, treatment options include tricyclic antidepressant (nortriptyline and desipramine), serotonin and norepinephrine reuptake inhibitors (SNRIs) (duloxetine and venlafaxine), calcium channel a2-*δ* ligands (gabapentin and pregabalin), opioids, and topical lidocaine [6]. Sodium channel blockers, such as carbamazepine and oxcarbazepine, are effective as first-line treatment for trigeminal neuralgia [7]. Pharmacotherapy alone provides a sufficient level of pain relief in about 30–40% of patients and previously common surgical interventions (such as neurotomies) have now been abandoned. Thus, there is a growing interest towards neurostimulation therapy for chronic pain [8]. An emerging tool for chronic pain treatment is transcranial focused ultrasound (tFUS), a noninvasive neurostimulation technique approved for thalamotomy in chronic neuropathic pain and for ablation of selected tumors. Many other applications of tFUS in neuropathic pain are still being investigated. In the present article, we propose an overview of approved and potential applications of focused ultrasound technology in pain management.

## 2. Pain Network

**Figure 1:**
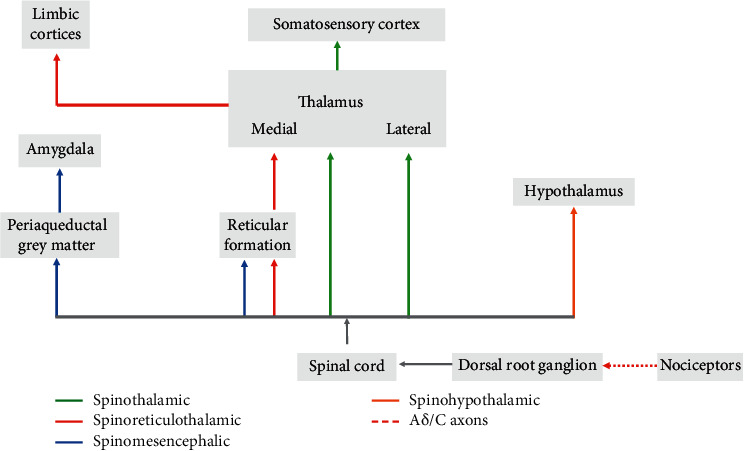
Central nervous system pain network.

Pain perception at the central nervous system level involves four ascending pathways, which convey nociceptive information from the dorsal root ganglions to the cerebral cortex (Figure 1). (1) The spinothalamic tract includes the axons of thermosensitive, nociceptive, and wide dynamic range neurons of the dorsal horn. The axons cross the midline, ascend in the anterolateral white matter, and terminate in both medial and lateral thalamic nuclei [9]. (2) The spinoreticular tract axons do not cross the midline and ascend in the anterolateral white matter of the spinal cord ending in the reticular formation, medial thalamus, and limbic cortices. (3) The spinomesencephalic tract ascends in the anterolateral quadrant of the spinal cord ending in the mesencephalic reticular formation, in the periaqueductal grey matter and in the amygdala, one of the most important nuclei of the limbic system. (4) The spinohypothalamic tract axons project to hypothalamic nuclei that act as autonomic control centers involved in the regulation of the neuroendocrine and cardiovascular responses to pain [9].

The thalamus contains several relay nuclei that participate in the central processing of nociceptive information [10]. For this physiological activity, two of the most important regions of the thalamus are the lateral and medial nuclear groups. The lateral nuclear group receives inputs through the spinothalamic tract and processes information about the location of the pain source, information usually elaborated from consciousness as acute pain. The medial nuclear group of the thalamus receives its major input from the spinothalamic tract and spinoreticulothalamic, including indirect connections through the reticular formation of the brainstem. Many neurons in the medial thalamus respond optimally to noxious stimuli and project widely to the basal ganglia and different cortical areas [9].

From the thalamus, information arrives at the somatosensory cortex (S1, S2, and supplementary sensorimotor area), the insula, and cingulate gyrus, defining a “body-self pain neuromatrix” [11]. It is characterized by anatomical distribution corresponding to the lesion, positive (painful or altered sensations) and negative symptoms (sensory deficit in the near area), altered sensation such as allodynia (pain due to a stimulus that does not provoke pain usually), and hyperalgesia (increased response to painful stimulation) [4, 6, 12].

Pain causes long-term modification of the central nervous system [13].

Oscillatory pathological brain activities in neurological disorders have been widely explored, in order to find biomarkers to target therapy [14, 15]. Neurophysiological studies, in pain, showed an abnormal bursting activity in the medial thalamus and in the central-lateral (CL) nucleus and a thalamocortical dysrhythmia that tended to normalize after CL thalamotomies [16–18]. These lesions could disrupt the synchronous, low-frequency activity in thalamocorticothalamic loop [19].

More recent evidence confirmed that, in patients with chronic neuropathic pain, an altered infraslow neural oscillatory activity is present in the medial thalamus and also in other regions of the somatosensory pathways, likely resulting from an abnormal neural-astrocyte coupling [20].

## 3. Neurostimulation Techniques for Chronic Pain Management

**Figure 2:**
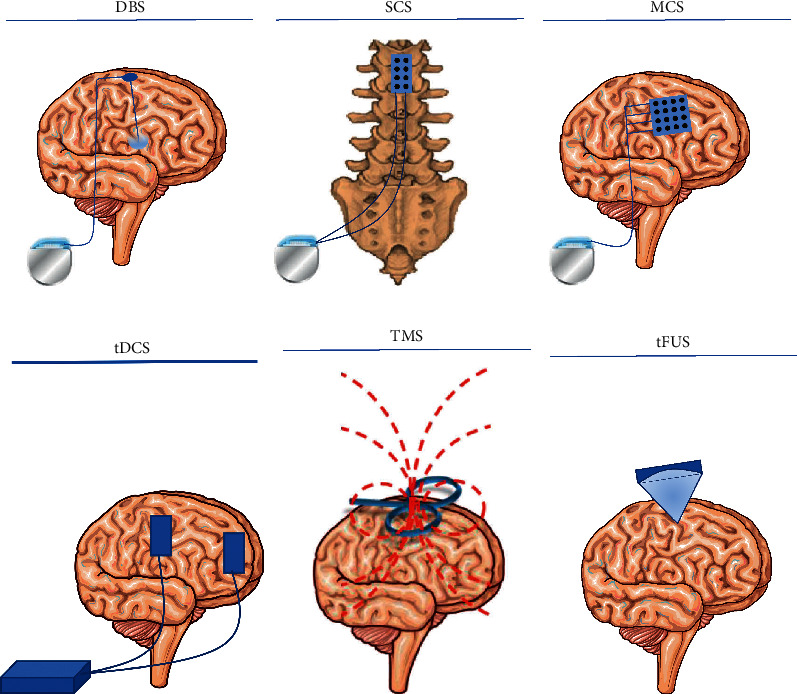
Neurostimulation techniques for chronic pain management. DBS = deep brain stimulation; SCS = spinal cord stimulation; MCS = motor cortex stimulation; tDCS = transcranial direct current stimulation; TMS = transcranial magnetic stimulation; tFUS = transcranial focused ultrasound stimulation.

Figure 2 shows the neurostimulation techniques for chronic pain management.

### 3.1. Invasive Neurostimulation

Deep brain stimulation (DBS), in last decades, has been widely used in the routine clinic for the treatment of movement disorders [21–26], epilepsy [27, 28], and obsessive-compulsive disorder [29], and exploratory studies showed that DBS targeting the thalamus (ventral posterolateral (VPL) nucleus or ventral posteromedial (VPM) nucleus), periventricular grey or periaqueductal grey, or anterior cingulate cortex might have a role in pain control [30, 31]. Another invasive technique, which showed promising results, is motor cortex stimulation [31–33]. After the first exploratory study by Shealy et al. [34], further evidence was provided supporting the efficacy of spinal cord stimulation in pain management, and currently, this is the most used neuromodulation technique for the management of chronic, intractable pain of limbs or trunk [35, 36]. Probably according to the gate control theory, it modulates pain signals through low-intensity electrical stimulation [37]. Spinal cord stimulation is mostly used for the treatment of failed back surgery syndrome and of complex regional pain syndrome, in combination with pharmacologic therapy and it received a weak recommendation in European Academy of Neurology (EAN) current guidelines [38, 39].

### 3.2. Noninvasive Neurostimulation

Noninvasive brain stimulation is a therapeutic approach alternative to invasive brain stimulation that has been explored for the treatment of neuropathic pain. Brain stimulation techniques primarily seek to modulate activity in the specific brain involved in pain processing reducing pain through interference with the ongoing neural activity in these areas [40].

Currently, such treatment options are limited to patients who do not respond to pharmacological treatments or have preexisting comorbidities that render pharmacological treatment at risk [41]. The main noninvasive brain stimulation techniques that showed a benefit in the treatment of neuropathic pain are repetitive transcranial magnetic stimulation (rTMS) and transcranial direct current stimulation (tDCS).

Meta-analysis of rTMS studies versus sham for pain intensity at short-term follow-up (0 to <1 week after intervention) (27 studies, involving 655 participants) demonstrated a small effect with heterogeneity [40]. One of the main issues regarding the use of rTMS of chronic pain treatment is the too short-lasting effect. Indeed, high-frequency M1 rTMS studies were initially based on single sessions, which produced delayed analgesic effects (by 2–4 days) lasting only 6–8 days [42]. However, for therapeutic purposes, a maintenance treatment (i.e., additional rTMS sessions performed at regular intervals) is required [43]. Evidence-based guidelines on the therapeutic use of rTMS, showed a Level A evidence (definite efficacy) for high-frequency (HF) rTMS of the primary motor cortex (M1) contralateral to the painful side for neuropathic pain and a Level B evidence (probable efficacy) for HF-rTMS of the left M1 or dorsolateral prefrontal cortex (DLPFC) for improving pain in fibromyalgia [44].

Weak recommendations are provided for the use of M1 rTMS in neuropathic pain and there are inconclusive recommendations regarding rTMS of the DLPFC in neuropathic pain [45]. For neuropathic pain, an rTMS stimulation scheme is to apply the stimulation contralaterally to localized neuropathic pain or on the left hemisphere in widespread neuropathic pain, using high frequency (5 Hz or more) stimulation, and a figure-of-eight coil oriented parallel to the midline over M1 for at least 1 week with at least 1000 pulses per session [46]. Increasing the total number of pulses per session and repeating the sessions for several days or weeks might enhance rTMS analgesia [45].

Anodal tDCS increases the excitability of the underlying cortex whereas cathodal tDCS decreases it [47] and a minimum duration of 5 min stimulation is needed to produce biological effects. This technique has been studied at the M1 and DLPFC level for the treatment of neuropathic pain. M1 stimulation reduces the thalamic and brainstem nuclei hyperactivity underlying pain [48], while DLPFC stimulation probably mediates analgesic effects by modulating affective-emotional networks related to pain [45]. A recent Cochrane library review found that tDCS may reduce pain when compared with sham but does not improve disability [40]. A weak positive recommendation for the use of tDCS in peripheral neuropathic pain is provided from EAN guidelines on central neurostimulation therapy in chronic pain conditions [45]. Safety is generally excellent, the main side effect of tDCS being a transient skin reaction below the stimulating electrodes [45].

## 4. Focused Ultrasound Neuromodulation

Transcranial focused ultrasound (tFUS) is a new tool for noninvasive neuromodulation [49]. Compared to classic noninvasive brain stimulation techniques, like magnetic or electric stimulations, tFUS can stimulate deep structures showing a higher spatial resolution. Thanks to this feature, tFUS can target, virtually, any site of the peripheral or central nervous system [49]; therefore, it is a perfect candidate as a tool for pain neuromodulation. In addition, tFUS allows a wide spectrum of stimulation parameters, which leads to different biological effects: from reversible neural activity facilitation or suppression (low-intensity, low-frequency ultrasound (LILFUS)) to irreversible tissue ablation (high-intensity focused ultrasounds (HIFU)) [49].

### 4.1. HIFU for Pain Management

Although neuromodulatory approaches have replaced many neurosurgical interventions in the management of chronic pain, ablative surgery remains an important part of the therapeutic approach for selected patients [50]. It is important to note that to date only FUS-mediated thalamotomy for chronic neuropathic pain and FUS-mediated ablation of selected tumors (bone metastases, osteoid osteoma, uterine fibroids, breast fibroadenoma, and pancreatic cancer) have been approved for human applications. All the remaining is represented by potential applications at a research stage. In Table 1, the most relevant studies on HIFU pain management are listed, with targets on central or peripheral nervous system.

#### 4.1.1. HIFU at the CNS Level

Among the different potential pain networks' targets (Figure 1), in the central nervous system to date, the approved indication in Europe for MR-guided HIFU is the thalamus for chronic neuropathic pain treatment.

*(1) HIFU Thalamotomy*. Neuropathic pain (NP) is pain arising as a direct consequence of a lesion or disease affecting the somatosensory system [152]. It has an estimated prevalence of 7–10% in the general population, it can have multiple central and peripheral etiologies, and its pathophysiology has not been fully clarified [37]. Management of NP is complex, and many patients do not respond to pharmacologic treatment [153]. For selected patients with refractory NP, alternative interventional strategies are considered, which include nerve blocks, surgical procedures that deliver drugs to desired areas, neuromodulation, and, less frequently, ablative procedures. However, controversies about these interventions exist [37, 38].

The posterior part of the CL nucleus, defined according to the Morel Stereotactic Atlas [154], has been proposed as a key target for pain management. However, placing proper lesions in CL nucleus is difficult, due to the complex three-dimensional structure of the nucleus [16].

Following a consolidated experience in stereotactic radiofrequency intracranial ablative procedures, the group from the Department of Functional Neurosurgery of the University Hospital of Zurich, Switzerland, used the HIFU technology to obtain the first noninvasive medial thalamotomies in patients suffering from chronic neuropathic pain. Preliminary results were published in 2009 [51] and extended results in 2012 [52]. Overall, 12 patients were treated (aged 45–75 years old), suffering from different types of neuropathic pain (facial, thoracic, lower extremity, upper extremity, and hemi body) of central or peripheral origin. Thermolesions were obtained in 11 over 12 treated subjects. CL thalamotomy was centered at the posterior part of the CL nucleus of the thalamus. Lesions were bilateral in 6 patients, and unilateral (contralateral to pain location) in 5 patients. Four patients had been previously treated with radiofrequency. Two patients had too small lesions. Therefore, analysis of global pain relief was reported for 9 patients only (8 patients at 1-year follow-up). Mean group pain relief was 71% at 2 days after treatment (9 patients), 49% at 3 months (9 patients), and 57% at 1 year (8 patients). VAS improvement was similar at 3 months (42%) and 1 year (41%). One patient had a focal bleed in the target site, associated with motor thalamus ischemia. All patients presented transient somatosensory, vestibular, or vegetative effects during sonication. In 8 patients, EEG recordings were carried out at baseline, 3 months, and 12 months, revealing a progressive reduction towards normal values of the spectral power amplitudes.

Data about the safety and efficacy of noninvasive HIFU mediated CL thalamotomies should be interpreted with caution, due to the small and heterogeneous population treated. Long-term clinical efficacy and long-term radiological evolution of the lesions should be assessed. Two clinical trials on MRgHIFU-mediated medial thalamotomies are ongoing: one (phase 1, single arm) including patients with chronic neuropathic pain due to radiculopathy, spinal cord injury, and phantom limb pain (NCT03111277). The other one (randomized, crossover, sham-controlled) is recruiting patients with chronic trigeminal neuropathic pain (NCT03309813). A global, multicenter, open-label, observational registry for data related to thalamotomy and pallidotomy procedures in multiple neurological diseases has been created (NCT03100474).

*(2) HIFU at Spinal Cord Level*. Another region of the CNS to which HIFU has been applied is the spinal cord (single preclinical study). Interventional approaches at the spinal cord level are considered in selected cases of refractory chronic neuropathic pain.

Destructive interventions aimed at interrupting selected spinal pain pathways are technically risky and are considered in a few selected cases. They include cordotomy (lesion of the lateral spinothalamic tract), trigeminal tractotomy at the C1 level, and extralemniscal myelotomy. Such interventions can be performed with minimally invasive stereotactic procedures [155].

In the early stages of FUS development, Shealy and Hanneman stimulated invasively spinal cord in animals, obtaining reversible effects on spinal reflexes [156]. Later, a specific study for pain was carried out. Following some evidence of pain relief after invasive neurosurgical procedures, discrete HIFU mediated spinal commissurotomies were performed in cats. At that time, a preliminary laminectomy was technically required. A partial reduction in gamma, delta, and pain-related C fibers-evoked potentials was observed [53].

#### 4.1.2. HIFU at the PNS Level

When targeting the peripheral nervous system (PNS), HIFU can be MR or US guided, depending on the target, the device, and the procedure.

Since the earliest stage of FUS development, experimental data are consistent with the hypothesis that FUS can induce a reversible or irreversible peripheral nerve ablation depending on doses. Lele [139] showed that some effects of FUS were temperature dependent and that the threshold between ablation and reversible effects could be narrow [139].

Other early studies on small animal models showed that FUS can selectively target C fibers while leaving A fibers relatively unaffected [140, 141].

Then, Foley et al. [142–144], in animal studies, observed that, depending on parameters, FUS treatment could induce a range of effects on nerves, going from temporary to complete conduction block acting on myelin with multiple mechanisms, with histological evidence of axonal demyelination and necrosis of Schwann cells [142] or on axons lesioning these structures, with histological evidence of axon degeneration [144]. They discussed how this property of FUS may be beneficial for patients with different severities of spasticity and pain [142–144].

Also, other studies on small animal models showed a range of possible FUS effects, on normal [145] and neuropathic (diabetic) peripheral nerves [146, 147], depending on stimulation parameters. Incidental findings of FUS reversible effects on peripheral nerves come from other applications, for example, reversible vocal cord paresis following HIFU treatment on thyroid nodules [157] or transient neuropathies after bone metastases treatment (REF). However, the mechanisms underlying the effects of FUS on tissues are still only partially understood [158]. Further preclinical research is important to clearly understand them before application in humans.

The major application of ablative HIFU at the PNS level is for pain relief in bone metastases. For this indication, MRgHIFU technology received both, the CE mark and the FDA approval. Phase III and phase IV clinical trials are ongoing. The other approved indication is the treatment of low back pain due to facet joint osteoarthritis. Further applications are in the research stage:

*(1) Cancer-Related Pain*. Cancer pain has a high prevalence among cancer patients and cancer survivors [159, 160]. Multiple causes contribute to its origin and persistence (including chemotherapy and surgery-related pain) and different types of pain (nociceptive, visceral, neuropathic, and incident cancer pain) can coexist [161]. Management of cancer pain is complex and associated with adverse effects, and often it is only partially successful [161]. The persistence of pain has a significant negative impact on the quality of life, morbidity, and mortality of cancer patients and can interfere with the therapeutic management (e.g., requiring reduced doses of chemotherapy) [162].

HIFU is being applied in many cancer-related painful conditions [163]. Following the experience on uterine fibroids [164]—frequent benign tumors that can cause pelvic pain [165]—HIFU has been applied to the palliative treatment of bone metastases, a frequent and multifactorial cause of cancer pain [166].

Mechanisms by which FUS induces analgesia in bone metastases are not entirely understood. Periosteal denervation and tumor debulking (alone or in combination) have been suggested [54]. A decrease in circulating immunosuppressive cytokines after MRgHIFU treatment [167, 168] has also been reported. However, its significance is still unknown. Procedure-related pain, skin burns, posttreatment fractures and neuropathy have been reported among the side effects of HIFU treatment of bone metastases [55]. The safety and efficacy profiles, the potential to perform multiple repeated treatments (in contrast with radiation therapy) in an outpatient modality make this approach extremely promising. MR-guided HIFU is now recommended as a second-line treatment for palliation of pain related to nonspinal and nonskull bone metastases after the failure of radiation therapy and it can be used as a first-line treatment when radiation therapy is contraindicated or the patient refuses it [163].

HIFU is also being applied with encouraging results to the palliative treatment of nonresectable pancreatic cancer [169, 170], which is a cause of pain in about 80% of cases [171].

To date, HIFU received both the CE mark and the FDA approval for the treatment of uterine fibroids and bone metastases. Furthermore, it has the CE mark for the treatment of osteoid osteoma and pancreatic cancer.

*(2) Nontumoral Bone/Joint Disease*. MRgHIFU is approved in Europe for the treatment of facet joint osteoarthritis. HIFU has been successfully applied in humans also to the knee joint. Preclinical research is ongoing on the sacroiliac joint. Studies on HIFU-mediated intervertebral disc nucleolysis are ongoing (cf. below).

*(3) Facet Joint Arthritis*. Facet joint arthritis is a common cause of low back pain and disability. Facet joints (or zygapophyseal joints) are innervated by the medial branches of the dorsal primary branch of the spinal nerves. Nociceptive stimuli can arise from mechanical factors and synovial inflammation and are often associated with a reflex painful muscular spasm of paraspinal muscles [172].

HIFU sonication is thought to induce a thermal ablation of the nerve terminals on the facet joints, although noninvasively. This application of HIFU could reduce complication risks and could be applied for outpatient pain management. Weeks et al. [119] published the results of a phase I, single-arm, open-label, prospective clinical trial. They treated 18 patients (mean age 48.2 years) affected by chronic back pain from facet joint arthritis. 13 patients were included in the follow-up. A significant improvement in pain scores and functional disability measures was observed. Results at 6 months were considered comparable to RF denervation. No adverse side effect was reported [119]. This trial provides preliminary data about the safety and efficacy of MRgFUS application in the treatment of low back pain due to facet joint osteoarthritis. For this indication, the ExAblate system (InSightec) received the CE mark. Preclinical research is also ongoing to address some technical issues [173].

A different approach targeting nerve endings on the facet joints has been recently explored at a preclinical level. Kaye et al. [120] demonstrated that, similar to radiofrequency neurotomy, a direct MRgHIFU ablation of the medial branch nerve can be achieved. Histology showed a clear nerve thermal necrosis without damage to adjacent structures [120]. A single-arm, open-label RCT is ongoing (NCT03321344, using Neurolyser XR portable device, FUSMobile Inc.).

*(4) Sacroiliac Joint Dysfunction*. Sacroiliac joint pain has an estimated prevalence of about 25% among patients with low back pain [174]. When conservative management has failed, interventional treatments for pain relief are considered. They include intra-articular injections, periarticular injections, sacral branch blocks, radiofrequency ablation of the supplying nerves, and minimally invasive fusion surgery [175, 176]. The sacroiliac joint is innervated by the dorsal branch of L5 and S1–S4 roots. However, individual variability exists [177]. This anatomical variability could give rise to incomplete radiofrequency ablation. For its unique noninvasiveness and high imaging definition, MRgHIFU-mediated ablation has been considered a potential additional strategy to explore. To date, one preliminary experiment has been performed in a swine model, as a proof of concept [121].

*(5) Spinal Disc Herniation*. The use of HIFU for the treatment of intervertebral disc herniation as a potential alternative strategy to percutaneous electrothermal treatment has been explored. Preliminary data about the feasibility of HIFU mediated approaches have been published for in vitro [122], ex vivo, and invasive in vivo models [123].

*(6) Knee Osteoarthritis*. Following the evidence of successful pain relief obtained by MRgHIFU-mediated treatment of bone metastases and facet joint arthritis, Izumi et al. [124] performed MRgHIFU-mediated knee treatment in 8 subjects affected by chronic pain from knee osteoarthritis. Sonication targeted the bone surface, below the rim osteophyte of the medial tibial plateau. The hypothesized mechanism of action is a denervation effect; 6 out of 8 patients had an immediate and significant VAS reduction, which was still present at 6-month follow-up in 4 subjects. No adverse side effects were reported [124].

Moreover, low-intensity FUS has been applied to knee osteoarthritis in a prospective randomized placebo-controlled clinical trial [148]. 106 patients were treated with “FLIPUS” (focused low-intensity pulsed ultrasound) + diclofenac sodium sustained-release tablets (53 patients with real FLIPUS, 53 with sham, all with diclofenac). FLIPUS stimulation was applied to both sides of the knee, for 20 min, once daily, for 10 days. The primary outcome was knee pain on movement for 5 minutes, assessed by VAS. The real stimulation group had a significantly greater improvement in VAS scores compared to the sham stimulation group. The improvement lasted for about 4 weeks after treatment. No FLIPUS-related adverse events were reported. In animal models, FLIPUS showed to promote bone regeneration [178] and extracellular matrix production through a downregulation of chondrocyte apoptosis, of the joint effusion volume, and of the release of prostaglandin E2 and nitric oxide [179].

Due to the high prevalence and socioeconomic impact of knee osteoarthritis, both approaches seem promising and deserve further research.

#### 4.1.3. Other Potential HIFU Applications at the PNS Level

*(1) Phantom Limb Pain*. Phantom limb is a heterogeneous disorder, with an estimated prevalence of 43%–51% in amputee and is frequently associated with phantom limb sensations [180, 181]. The causes of variable individual susceptibility are not known. Stump neuromas, which contain disorganized A fiber and C fiber terminals, are frequently associated with a phantom limb. The central nervous system is also involved: pathological phenomena at the spinal cord level (sprouting of fibers from lamina III and IV in lamina II, increased excitability, and expansion of the dorsal horn receptive field) and at a supraspinal level (thalamus, sensorimotor cortex) have been described. Surgical invasive procedures are limited to selected refractory cases and include neuroma ablations, dorsal root entry zone lesioning, anterolateral cordotomy (of the spinothalamic tract), thalamotomy, and sympathectomy. However, pain relief produced by these procedures is often short-lived [182]. Transected nerve endings in residual limbs of amputee patients have been found more sensitive to HIFU sonication compared to control tissue and intact nerves [183]. Preclinical studies had already shown similar data, starting from pioneering studies [140, 141]. This higher sensitivity of transected nerve endings to FUS stimulation could allow selective denervation. A clinical trial is ongoing (single group, open-label, NCT03255395) to assess feasibility, safety, and efficacy of MRgHIFU-mediated ablation of stump neuromas (also trial NCT03111277 recruits patients suffering from phantom limb pain, which will be treated by MRgHIFU-mediated CL thalamotomy). However, data about the safety and long-term efficacy are needed.

*(2) Ablation of Peripheral Nerves*. FUS is being studied as a potential noninvasive option for producing peripheral nerve ablations.

Peripheral nerve ablation is a technique used in some cases of chronic neuropathic pain and cancer pain [37]. It is also used for patients suffering from low back pain, to predict the outcome of ablative procedures (see above). Ablative methods include chemical denervation, cryoneurolysis, and radiofrequency ablation. In swine models of intercostal nerves ablation, HIFU mediated lesions showed a well-demarcated thermal necrosis immediately after the procedure. At the same time, neither RF ablations nor alcohol ablations showed radiological signs of lesions, suggesting a different mechanism of action of FUS [125].

The feasibility of MRgHIFU-mediated ablations was demonstrated in other large animal studies for the sciatic nerve [126] and the lumbar medial branch nerve [120]. Some difficulties exist in visualizing superficial peripheral nerves with MR. In a pilot study, 3D MR neurography showed high potential for guiding HIFU therapy ablation of peripheral nerves [127]. An ultrasound-guided approach for HIFU peripheral nerve block has been demonstrated feasible in an in vivo large-animal study. However, at the current stage, only MR-guided procedures allow a noninvasive temperature monitoring strategy. A noninvasive thermometry technology for ultrasound-guided ablation is at a developmental stage [128].

### 4.2. LILFUS for Pain Management

There is increasing interest in the biological reversible effects of FUS on targeted tissues [49]. Therefore, many researchers are trying to better understand the neurological and histological effects of FUS stimulation using different parameters. Notably, FUS applications at nonablative doses are all currently at a research stage and some of them are only speculative (Table 2).

*(1) Targeted Drug and Gene Delivery through the Blood-Brain Barrier*. FUS could control pain through different mechanisms such as increasing Blood-Brain Barrier permeability, increasing the concentration of central acting analgesic drugs in CNS, or modulation of the expression of genes involved in pain perception. To the best of our knowledge, no specific studies are exploring these approaches. Therefore, these FUS applications for pain relief are purely speculative.

*(2) Assessment of Pain Thresholds and Study of Pain*. Gavrilov et al. [129, 130] described that FUS stimulation of the human hand could elicit different sensations (tactile, temperature, and pain) depending on the stimulation and environmental parameters. Following these observations, Wright et al. [131, 132] recorded and described brain evoked potentials from stimulation of deep nociceptors in the proximal interphalangeal joint of the index finger. The evoked potential waveform was reproducible and correlated with the subjective evaluation of the painful stimulus measured by visual analogue scales. It became apparent that focused ultrasound could have been used to obtain an objective measure of the perceived pain [131, 132]. More recently, it has been confirmed that FUS can generate reliably sensed, cutaneous sensations in humans, mediated by mechanoreceptors [133].

Other recent preclinical evidence from animal models suggested that FUS could be used to diagnose patients with neuropathies: in a rat model of neuroma, FUS preferentially stimulated neuropathic tissue with a high spatial resolution [135]; in an animal model of sciatic neuropathic pain, FUS stimulated preferentially neuropathic tissue [136]; and in another preclinical study, FUS preferentially stimulated inflamed subcutaneous tissue [137]. Furthermore, since inflammation-based pain diseases present circadian rhythms with a diurnal variation of pain, FUS could be used for studying chronotherapeutic pain management: in a rat model of inflammatory pain, the threshold of pain assessed by pulsed FUS displayed diurnal variations, with a higher threshold during the night [138].

It has also been found in human studies that FUS preferentially stimulates transected nerves within residual limbs [183] and that FUS can induce sensations in the selected structure of a pathological rotator cuff [134]. Those findings suggest that FUS could potentially help physicians identify deep sources of pain and assessing the peripheral versus central source of pain.

*(3) Other Potential Applications of LILFUS in PAIN*. Monteith et al. [150] demonstrated in a cadaveric and laboratory model that trigeminal nerve could be targeted noninvasively by a MRgFUS system at the root entry zone level without causing appreciable heating of critical surrounding brain structures. Through a specific combination of power and sonication duration, they delivered a lower temperature compared to that used in lesional FU surgery. This dosage of sonication could hypothetically induce demyelination and interruption of the pain fibers only in vivo, without causing necrosis of the entire nerve. However, some concerns remain about the heating of the internal acoustic canal [150]. To date, no in vivo studies have been performed. According to recent guidelines, the quality of evidence about interventional management of trigeminal neuralgia is low, and the strength of recommendation is inconclusive. Microvascular decompression only may offer the longest duration of pain control [38].

Preliminary work on a chronic migraine rat model suggested that pulsed, high-intensity focused ultrasound targeting the occipital nerve could be a noninvasive treatment for chronic migraine even at nonablative intensities [151]. A convergence of nociceptive information from meningeal afferents and cervical afferents has been demonstrated in the great occipital nerve. This convergence could explain the benefit produced by great occipital nerve block in some forms of headache [184]. Regarding peripheral nerve blocks in primary and secondary headache, recent recommendations stated that, except for great occipital nerve block in cluster headache, there is limited evidence from controlled studies [185]. Concerning occipital nerve stimulation, the position statement of the European Headache Federation reported that occipital nerve stimulation must be employed with caution and only carefully considered for the most severely affected patients with medically refractive cluster headache. Occipital nerve stimulation demonstrated preventive effects, except in a case series of migraine patients which showed acute effects, with headache suppression within 30 min from switching on the stimulation, and a headache recurrence with a peak within 20 min from switching off the stimulation [186, 187].

## 5. Conclusions

Focused ultrasound technology is opening new scenarios in the field of pain management. Nonablative FUS has huge research potential. Noninvasive reversible modulations of deep pain pathways with a high spatial resolution can be performed. A few examples of potential applications include obtaining objective measures of pain, three-dimensional mapping of CNS pain regions, localizing deep sources of pain, identifying the peripheral contribution in chronic painful conditions of mixed origin, noninvasively predicting the outcome of ablative procedures, and obtaining acute pain relief through reversible conduction blocks. At the current stage of development, priority should be given to a better understanding of the mechanisms of action and biological effects at a preclinical level, along with explorative safety studies for human applications.

Ablative FUS strategies are successfully expanding the therapeutic options available for chronic cancer pain treatment and low back pain. Other ablative FUS applications seem promising and their development should be encouraged. The noninvasiveness of FUS technology is a unique characteristic. However, we believe that any new approach, regardless of how complex or expensive it is, would be ineffective if its application is not rationally guided by the knowledge of underlying pathophysiology and by the appropriate selection of patients to be treated. Pursuing high standard research and high standard clinical management in the field of pain is necessary. Long-term results, on large series of patients from randomized controlled clinical trials and with standardized outcome measures, are needed.

## Figures and Tables

**Table 1:** HIFU for pain management.

Site	Guide	FUS application	Target	Ongoing clinical trials	Published results	Stage
*CNS*	MR	Chronic neuropathic pain	Thalamus (CL nucleus)	NCT03111277	[51,52]	*Human* ¶
	MR	Chronic pain from spinal cord injury				
	MR	Phantom limb pain				
	MR	Chronic trigeminal neuropathic pain	Medial thalamus	NCT03309813		
	NA	Chronic neuropathic pain	Spinal commissurotomy	—	[53]	*Preclinical (not further developed)*

*PNS*	Management of tumor-related pain					
	MR or US	(1) *Bone metastases* and primary bone malignancies related pain	Lesion	NCT02616016 NCT02718404 NCT01833806 NCT03106675 NCT00981578 NCT01091883 NCT02076906	[54–67]	*Human ¶, §*
	MR or US	(1) *Osteoid osteoma* and other benign bone tumors related pain	Lesion	NCT02618369 NCT02923011	[68–75]	*Human ¶*
	MR or US	(1) *Uterine fibroids* related pain	Lesion	NCT02736435	[76–93]	*Human ¶, §*
	MR or US	(1) *Breast fibroadenoma* related pain	Lesion	NCT02488655 NCT03044054	—	*Human ¶*
	MR or US	(1) *Pancreatic cancer*-related pain palliative treatment	Lesion	NCT01786850 (expanded acc.) NCT00637364 (suspended)	[94–114]	*Human ¶*
	NA	(1) Recurrent rectal cancer-related pain palliative treatment	Lesion	NCT02528175	—	*Human*
	NA	(1) Recurrent gynecological cancer-related pain palliative treatment	Lesion	NCT02714621	[78]	*Human (case report)*
	NA	(1) Advanced rectal and gynecological cancers-related pain palliative treatment	Lesion	NCT01097239	—	—
	Other nontumoral gynecologic conditions					
	US	(1) Endometriosis related pain	Lesion	—	[103, 115–117]	*Human*
	US	(1) Adenomyosis related pain	Lesion	—	[118]	*Human*
	Management of bone/joint nontumoral pain					
	MR	(1) Low back pain from lumbar *facet joint osteoarthritis* (without radiculopathy)	Facet joint/lumbar medial branch nerve	NCT03321344	[119, 120]	(a) Human ¶ (b) Preclinical
	MR	(1) Low back pain from lumbar sacroiliac joint dysfunction	Sacroiliac joint	—	[121]	Preclinical
	NA	(1) Spinal disc herniation related pain	Intervertebral disc	—	[122, 123]	Preclinical *(not further developed)*
	MR	(1) Knee osteoarthritis related chronic pain	Knee joint	—	[124]	Human
	Other					
	MR	Phantom/residual limb pain	Stump neuromas	NCT03255395	—	Human
	MR or US	Chronic neuropathic pain (nerve ablation, irreversible conduction block)	Peripheral nerves	—	[120, 125–128]	Preclinical

¶ = CE marked; § = FDA approved. CL = central lateral, CNS = central nervous system, CT = clinical trial, FUS = focused ultrasound, HIFU = high-intensity focused ultrasound; MRg = magnetic resonance-guided, NA = not applicable, USg = ultrasound-guided, and PNS = peripheral nervous system

**Table 2:** LILFUS for pain management.

Site	Guide	FUS application	Target	Ongoing CT	Published results	Stage
*CNS*	MR	*Functional mapping of cerebral pain networks*	Brain	—	—	†
	MR	*Targeted drugs and gene delivery through the Blood-Brain Barrier for pain relief*	Blood-Brain Barrier	—	—	†
	MR or US	*Tissues targeted delivery of temperature-sensitive drugs for pain relief*	Target tissue	—	—	†

*PNS*	US	Assessment of pain thresholds: (1) Diagnosis of neuropathies (2) Evaluation of effectiveness of analgesics (3) Study of the diurnal variation of pain (4) Localization of deep sources of pain	Peripheral nerves, skin, deeper structures	—	[129–134] *Preclinical*:[135–138]	*Human studies and preclinical*
	MR or US	Chronic neuropathic pain, spasticity-associated pain (reversible conduction block/pain fiber selective ablation)	Peripheral nerves	—	[139–147]	Preclinical
	No	Knee osteoarthritis related chronic pain	Knee joint	—	[148]	Human
	No	Noninvasive stimulation of acupoints	Skin	—	[149]	Human
	MR	Chronic trigeminal neuralgia	Trigeminal root entry zone	—	[150]	*Cadaveric and in vitro model*
	NA	Chronic migraine	Occipital nerve	—	[151]	*Preclinical*

† = speculative application (no studies on pain ongoing currently). CL = central lateral, CNS = central nervous system, CT = clinical trial, FUS = focused ultrasound, LILFUS = low-intensity, low-frequency ultrasound; MRg = magnetic resonance-guided, NA = not applicable, USg = ultrasound-guided, PNS = peripheral nervous system.

## Data Availability

No data were used to support this study.
